# EEG Source Connectivity Analysis: From Dense Array Recordings to Brain Networks

**DOI:** 10.1371/journal.pone.0105041

**Published:** 2014-08-12

**Authors:** Mahmoud Hassan, Olivier Dufor, Isabelle Merlet, Claude Berrou, Fabrice Wendling

**Affiliations:** 1 INSERM, U642, Rennes, France; 2 Université de Rennes 1, LTSI, Rennes, France; 3 Télécom Bretagne, Institut Mines-Télécom, UMR CNRS Lab-STICC, Brest, France; IIT - Italian Institute of Technology, Italy

## Abstract

The recent past years have seen a noticeable increase of interest for electroencephalography (EEG) to analyze functional connectivity through brain sources reconstructed from scalp signals. Although considerable advances have been done both on the recording and analysis of EEG signals, a number of methodological questions are still open regarding the optimal way to process the data in order to identify brain networks. In this paper, we analyze the impact of three factors that intervene in this processing: i) the number of scalp electrodes, ii) the combination between the algorithm used to solve the EEG inverse problem and the algorithm used to measure the functional connectivity and iii) the frequency bands retained to estimate the functional connectivity among neocortical sources. Using High-Resolution (hr) EEG recordings in healthy volunteers, we evaluated these factors on evoked responses during picture recognition and naming task. The main reason for selection this task is that a solid literature background is available about involved brain networks (ground truth). From this a priori information, we propose a performance criterion based on the number of connections identified in the regions of interest (ROI) that belong to potentially activated networks. Our results show that the three studied factors have a dramatic impact on the final result (the identified network in the source space) as strong discrepancies were evidenced depending on the methods used. They also suggest that the combination of weighted Minimum Norm Estimator (wMNE) and the Phase Synchronization (PS) methods applied on High-Resolution EEG in beta/gamma bands provides the best performance in term of topological distance between the identified network and the expected network in the above-mentioned cognitive task.

## Introduction

Neuroimaging techniques can be used to identify brain networks involved in normal brain functions (learning, memory, behavior adaptation to stimuli or emotions) as well as in neurological disorders like epilepsy, autism or schizophrenia [1]–[3]. In this context, functional MRI has considerably developed during the past two decades and is now commonly used to characterize brain connectivity [4]. In the meantime, a number of studies reported that electro-/magneto-encephalography (EEG/MEG) associated with appropriate signal processing techniques might also bring relevant information about normal networks activated during cognitive activity [5] or about disrupted networks associated for instance with tumors [6]).

Actually, over the last three decades, a large variety of methods aimed at solving the EEG/MEG inverse problem in order to localize and reconstruct the sources of brain activity have been proposed (see review in [7], [8]). Meanwhile, methods devoted to the characterization of functional [9]–[11] and effective ([12], see review in [13]) connectivity from EEG/MEG signals have considerably developed.

Interpretation of connectivity measures from sensor level recordings is not straightforward, as these recordings suffer from a low spatial resolution and are severely corrupted by effects of field spread [14]. To overcome these difficulties, several attempts to apply connectivity methods on the temporal dynamics of brain sources reconstructed from scalp EEG/MEG signals have been reported [5], [15]–[23], see [14] for review). This approach is conceptually very appealing as networks are directly identified in the source space, typically in the neocortex. However, it raises a number of methodological issues. First, it requires i) to solve the ill-posed EEG/MEG inverse problem. Second, a functional connectivity method must be chosen among the many available ones. Third, volume conduction effects can never be completely abolished in source space.

Intuitively, it is expected that the final result, i.e. the identified networks, will directly depend on the chosen information processing methods. Consequently, the central question raised here is related to the choice of the best combination of methods (EEG/MEG inverse problem solution + the functional connectivity estimation) which is likely to reveal the actual networks that activate during the considered brain process. In addition, in both steps of the signal processing procedure, some key parameters are also expected to influence the results such as the threshold applied to the adjacency matrices or the considered delay between the time courses of reconstructed sources, among others.

In this paper, we report a quantitative comparison of methods aimed at identifying brain networks from scalp EEG data. We focus on functional connectivity estimated from evoked responses [5], [17]. The novelty of the paper is threefold: first, the evaluation methodology is based on a well-controlled cognitive task (picture recognition and naming) for which a solid background is available regarding the topology of activated networks that are therefore used as a ground truth. Second, scalp recordings were performed using a high-resolution EEG (hr-EEG) system characterized by an excellent temporal resolution (1 ms) and by an improved spatial resolution (256 electrodes), as compared to more classical systems (32–64 electrodes). Third, the comparison consisted in a “two-dimensional” analysis allowing for quantifying the joint effect of the inverse and connectivity methods. In addition, we also addressed three corollary issues related to i) the impact of the number of scalp electrodes used to solve the inverse problem, ii) the influence of frequency bands in which the functional connectivity is estimated and iii) the contamination of functional connectivity measures by the leakage of sources.

The paper is organized as follows. The connectivity and inverse algorithms we retained for evaluation are described in the methods section along with details about the cognitive task performed by subjects. Results obtained from the quantitative comparison of analyzed methods are then presented. They show that fairly different networks are identified from the same EEG data set when different methods are being used. However, they also suggest that some combinations (inverse + connectivity methods) could lead to the identification of networks topologically close to the expected ones, as reported in the literature based on neuroimaging data recorded during the same cognitive task.

Our evaluation methodology involves three main aspects:

- The inverse algorithm used to estimate the cortical sources and reconstruct their temporal dynamics.
- The connectivity method used to assess statistically significant functional relationships between the temporal dynamics of sources.
- The cognitive task performed by the subject, which is supposed to activate relatively well-defined brain networks.

Regarding the first aspect, several approaches have been proposed to solve the inverse problem and these have been widely used in the context of brain source localization either in normal or pathological conditions. Among the methods especially designed for distributed brain sources, the most popular algorithms include (but are not limited to) the Minimum Norm Estimate (MNE) and its weighted version (wMNE) [24] [25], [26], Low resolution brain electromagnetic tomography (LORETA) and standardized low resolution brain electromagnetic tomography (sLORETA) [27], [28]. In addition, some efforts have been done to evaluate inverse algorithms in the view of localizing the brain sources in specific applications [29], [30].

Regarding brain connectivity methods applied to EEG/MEG, bi- or multi-variate approaches proposed so far can be divided into two main categories depending on the assumptions made about the statistical coupling between signals. The first category includes linear methods such as the linear cross-correlation (R^2^) [31] or the coherence function [32]. The second category includes nonlinear methods based on mutual information (MI) [33], nonlinear regression (*h* ^2^) [34], [35], generalized synchronization (GS) [36] and phase synchronization (PS) [37]. Recently, some efforts have been made to evaluate the performance of some of these measures in different scopes. An evaluation of the connectivity methods conducted on different synthetic and physiological models highlighted the high variability in the results provided by these methods, depending on the model that generates analyzed signals [9], [10]. Interestingly, a conclusion from these studies is that regression-based methods show the best performance, as opposed to more sophisticated methods which may be blind to coupling changes. Finally, methods were also reported to reduce the effect of source leakage on functional connectivity measures. The general idea is to assume that a zero (or even very low) time lag is likely to correspond to a “volume conducted” activity as opposed to a “functionally related” activity for which a delay is expected due to synaptic transmission for instance. A method based on the imaginary part of the coherence function, often referred to as Imaginary Coherence (ImC), was initially proposed by Nolte et al. [38] and was further improved regarding the bias inherent to the coherence estimation [39].

To our knowledge, very few attempts to evaluate the connectivity measures at the source level were reported so far. The work of David et al. [18] on functional connectivity in neural mass models showed that methods can detect coupling but with different sensitivity profiles depending on the frequency band (broad vs. narrow band) and on the type of the connection (linear vs. nonlinear) [18]. Reported results suggest that the PS methods are the most sensitive ones with regard to nonlinear couplings and that the MI method shows the highest sensitivity in broadband analysis. Another comparison between connectivity measures at source level was reported by Astolfi et al. but this study focused on effective connectivity [40]. For the purpose of this study, we retained five commonly -used functional connectivity methods based on R^2^, MI, ImC and two PS indexes.

A crucial parameter in functional connectivity estimation is the threshold that has to be defined to get the connectivity matrix. This factor affects directly the resulting network. As our main concern is to standardize the comparison between methods and as there is no consensus about the optimal way to set this threshold, we kept the 10% of the strongest functional connections for each method, as discussed in [41].

Finally, as far as the cognitive task is concerned, it is worth mentioning that picture recognition and naming has been a topic of large interest. Many studies were devoted to the identification of brain regions and/or networks activated during this task, mainly from fMRI and PET data. The main advantage of this task in the particular context of our study is that the activated network is relatively well described, and involves the bilateral occipito-temporal cortex, the left parietal, the left inferior temporal and bilateral inferior frontal cortices, as described in [42]–[49].

MEG-based analyses conducted in the same task revealed that the activation proceeds from the occipital cortex (<200 ms) to left parietal and bilateral temporal areas (>200 ms) and further to frontal regions (>400 ms) [50]–[54]. Using acute electro-stimulation techniques, Mandonnet et al. showed that the information flow is going from the left occipital to the left temporal region during picture naming [55].

A conclusion from this synthetic review of the literature is that no attempt has been made so far to evaluate the joint capacity of inverse/connectivity methods applied to EEG to disclose brain networks involved in a specific cognitive task. This evaluation is the main objective of our study in which some of the above-described methods are evaluated on EEG signals recorded during the picture recognition and naming task, as detailed in the next section.

## Materials and Methods

### Connectivity measures

For the purpose of the paper, we selected five methods that have been widely used to estimate functional brain connectivity from electrophysiological signals (local field potentials, depth-EEG or EEG/MEG).

#### A. Cross-correlation coefficient (R^2^)

This is one of most classical measures of interdependence between two time series. The cross-correlation coefficient measures the linear correlation between two variables X and Y as a function of their time delay (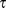). The linear correlation coefficient is defined as:
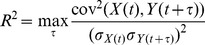
where 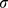 and *cov* denote the standard deviation and the covariance, respectively. R^2^ ranges from 0 (*X* and *Y* are independent) to 1 (*Y* is a linear function of *X*).

#### B. Mutual information (MI)

The information theory was proposed nearly 60 years ago [56]. The theory is based on the concept of entropy, which can be defined as the average total of information necessary to encode a discrete variable *X* with *M* possible outcomes *Xi* with probability *p_i_*. The Shannon entropy is then defined as:
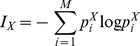



The mutual information (MI) between signal *X* and *Y* is defined as:
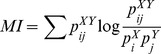
where 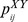 is the joint probability of *X* = *X_i_* and *Y* = *Y_j_*. If there is no relationship between them, 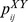 = 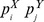, so that the MI is zero for independent processes. The mutual information computes the statistical dependence between *X* and *Y*, with no assumption about their generating process (linear or nonlinear). It can be shown that the estimation of MI is biased when some bins contain no point 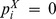. To overcome this difficulty we used the corrected algorithm initially proposed by Roulston et al. [57]. The same computation way of MI has been adopted by [18] which is different than [58] where a temporal embedding is used.

#### C. Phase synchronization (PS)

It is well known that the phases of two oscillators may synchronize even if their amplitudes stay uncorrelated [59]. The general principle of the phase synchronization (PS) method is to detect the existence of a phase locking between two systems defined as:
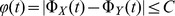
where Φ*_X_(t)*, Φ*_Y_(t)* are the unwrapped phases of the signals (*X* and *Y*) representative of the two systems at time bins *t* and *C* a constant. The first step for estimating the phase synchronization is to extract the instantaneous phase of each signal. Two different techniques can be used: the Hilbert transform and the wavelet transform. As the application of both approaches produces close results [58], we are using the method based on Hilbert transform in our study.

The second step is the definition of an appropriate index to measure the degree of synchronization between estimated instantaneous phases. Several measures have been proposed in the literature where two of them are selected in the paper. The first one [60] called the single-trial phase locking value (sPLV) defined as:
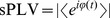
where 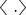 denotes average over time. The second phase synchronization measure is using the Shannon entropy 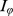 of the distribution of 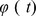. The entropy is normalized according to the maximal entropy 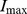 obtained in the case of a uniform distribution of the phase difference (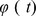 is random). The second measure [61] is called Phase Entropy (PE) and defined as:



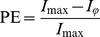



The two above-described indexes sPLV and PE have the same range of variation: they are close to 0 for uncorrelated signals, whereas they tend to 1 for strong phase synchronization.

#### D. Imaginary coherence (ImC)

The coherence (C) function gives the linear correlation between two signals *X* and *Y* as a function of the frequency. It is defined as their cross-spectral density function 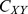 normalized by their individual auto-spectral density functions 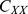 and 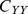. Nolte et al. [38] have showed that the use of the imaginary part of the coherence function can reduce the effect of the volume conduction. In this paper, we evaluate the imaginary coherence (Im*C*) defined as:
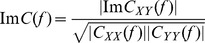



Signals are subdivided into overlapping segments and the corresponding spectra are averaged to estimate the (cross) spectral density functions (averaged periodogram, Welch’s method, overlapping = 50%).

### Frequency bands

Connectivity methods can be applied either on broad band or narrow band signals obtained from the filtering of raw signals, typically in the classical EEG frequency bands (delta, theta, alpha, beta and gamma). It has already been shown that the frequency parameter has a dramatic impact on the connectivity values [10]. In the present study, we restricted our analyses to two different frequency bands, namely beta (14–30 Hz) and low gamma (30–45 Hz). Indeed, these two bands are the most relevant ones in the context of the cognitive task performed by the subjects, as reported in [18], [62].

### EEG inverse problem

According to the linear discrete equivalent current dipole model, EEG signals 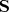
(t) measured from M channels can be expressed as linear combinations of P time-varying current dipole sources 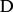
(t):
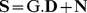
where 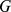 and 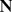
(t) are respectively the matrix containing the lead fields of the dipolar sources and the additive noise.

In the general case, the inverse problem consists in finding an estimate 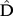
(t) of the dipolar source parameters (typically, the position, orientation and magnitude), given the EEG signals 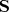
(t) and given the gain matrix 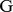. This matrix can be computed from a multiple layer head model (volume conductor) and from the position of electrodes. For instance, the Boundary Element Method is a numerical method classically used in the case of realistic head models.

As this problem is ill-posed (P>>M), physical and mathematical constraints have to be added to obtain a unique solution among the many solutions that minimize the residual term in the fitting of measured EEG signals. For instance, using segmented MRI data, the source distribution can be constrained to a field of current dipoles homogeneously distributed over the cortex [63], and normal to the cortical surface.

Technically, in the source model, we assumed that EEG signals are generated by macro-columns of pyramidal cells lying in the cortical mantle and aligned orthogonally with respect to its surface [64]. Thus, the electrical contribution of each macro-column to scalp electrodes can be represented by a current dipole located at the center of gravity of each triangle of the 3D mesh and oriented normally to the triangle surface. Using this source space, the so-called distributed approaches described below only estimate the moment of dipole sources.

#### A. Minimum Norm Estimate (MNE)

Minimum norm estimates [24], [65] are based on a search for the solution with minimum power using the L2 norm to regularize the problem. This type of estimators is well appropriate to distributed source models where the dipole activity is likely to extend over some areas of the cortical surface.
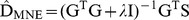
where I is the identity matrix and 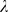 is the regularization parameter that weights the influence of priors in the solution.

#### B. Weighted Minimum Norm Estimate (wMNE)

The weighted MNE algorithm compensates for the tendency of MNE to favor weak and surface sources. This is done by introducing a weighting matrix 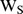:
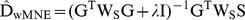
where matrix W_S_ adjusts the properties of the solution by reducing the bias inherent to MNE solutions. Classically, Ws is a diagonal matrix built from matrix G with non-zero terms inversely proportional to the norm of the lead field vectors.

#### C. Low resolution Brain Electromagnetic Tomography (LORETA)

In Low resolution electromagnetic tomography (LORETA) the main feature is to hypnotize that neighbor dipoles are strongly correlated. Therefore, a spatial smoothness constraint is explicitly promoted by applying a Laplacian operator to the sources in the regularization term. Moreover, as in wMNE, the columns of G are normalized to compensate for the misestimating of deep sources. In this method, for constrained number, position and orientation of dipolar sources, the estimate of the dipole moments is given by:
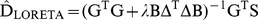
where B is a diagonal matrix for the column normalization of **G** and 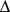 is a Laplacian operator.

#### D. Standardized low resolution brain electromagnetic tomography (sLORETA)

Despite its name, sLORETA [28] is not based on LORETA but rather on MNE. Indeed, sLORETA uses the source distribution estimated from MNE and standardizes it a posteriori by the variance of each estimated dipole source.
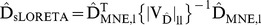
where 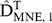 is the current density estimate at the *l*th voxel given by the minimum norm estimate and 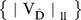 is the *l*th diagonal block of the resolution matrix 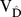 (variance of the estimated current density) defined as 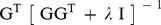. Therefore, contrarily to LORETA, MNE wMNE, sLORETA does not estimate intensity of a given source, but rather the probability of this source to disclose high amplitude as compared to the other ones.

LORETA and sLORETA inverse methods have been originally described using the whole brain volume as source space [28], [66]. For the present study, in order to ease the comparison with MNE and wMNE, we have implemented these methods by restricting the source space to the cortical surface.

The choice of 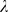 is important and many approaches have been proposed to estimate it. Although there is no agreement on any optimal solution [19], As the main purpose of our study is to compare different approaches based on both inverse solutions and connectivity estimates, we chose to limit the number of intrinsic factors. Consequently, we used the same value of 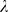 = 1 for the four inverse algorithms based on the signal to noise ratio of our signals. Note that the same threshold was applied to both intensity (MNE, wMNE, LORETA) and probability (sLORETA) approaches.

### Comparison method

**Figure 1:**
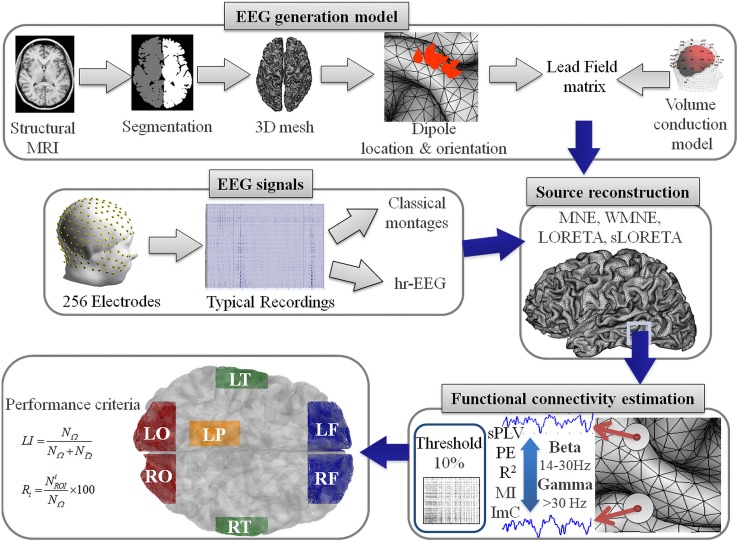
The different steps of the comparative study. **hr-EEG**: high-resolution EEG, **MNE**: Minimum norm estimate, **wMNE**: Weighted Minimum norm estimate, **LORETA**: Low resolution Brain Electromagnetic Tomography, **sLORETA**: Standardized Low resolution Brain Electromagnetic Tomography, **sPLV**: single-trial Phase Locking Value, **PE**: Phase Entropy, **R^2^**: linear correlation coefficient, **MI**: Mutual Information, **ImC**: Imaginary Coherence **ROIs**: Regions of Interest, **LO**: Left Occipital; **RO**: Right Occipital, **LT**: Left Temporal, **RT**: Right Temporal, **LF**: Left Frontal, **RF**: Right Frontal, **LP**: Left Parietal.

The different steps of the comparative analysis are summarized in figure 1. In step 1, the lead field matrix G is estimated using the BEM based on i) a high-resolution 3D mesh of the white/grey matter interface for the source model and on a realistically-shaped head model (3 layers) for the volume conductor, both obtained from MRI data segmentation.

**Figure 2:**
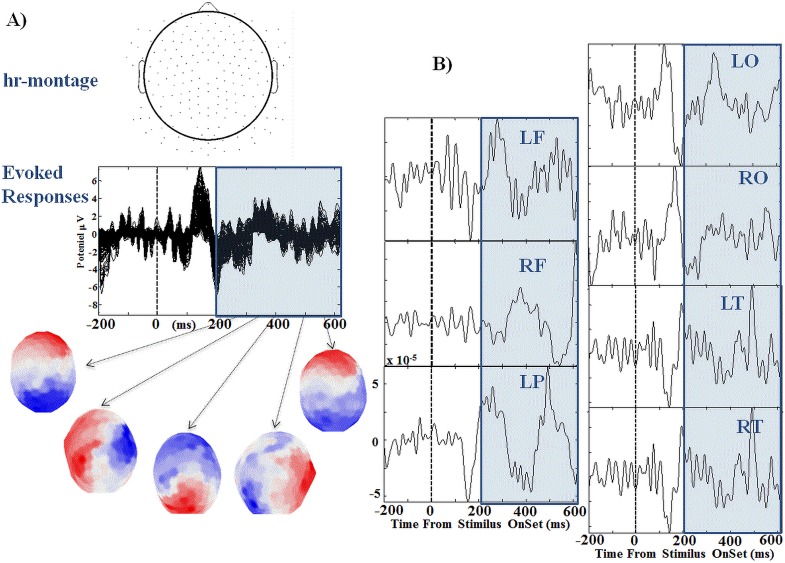
EEG signals and its reconstructed sources. A) The recorded evoked responses for a given subject, B) the corresponding reconstructed sources and C) an example of the sources in each of the ROIs. The window of analysis is illustrated in transparent blue rectangle.

In step 2, the temporal dynamics of dipolar sources 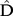
(t) are estimated from scalp EEG signals S(t) recorded during the picture recognition and naming task. An example of EEG signals and corresponding activation maps at different instants are shown in figure 2A. The activation maps show an argument about the concordance between our data and the state-of-the-art concerning the activated regions during the analyzed task. The figure shows also an example of the reconstructed sources in each of the ROIs (figure 2B). The window of analysis used to compute the functional connectivity is also presented.

The four above-described methods (MNE, wMNE, LORETA, sLORETA) are used to estimate 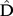
(t) with retaining only 10% of the highest energy sources. More precisely, the relative energy of each estimated source is calculated, these values are sorted in decreasing way and the sources corresponding to the highest 10% are retained. This calculation has been done on the whole window and then applied to all time points.

Four montages were used to analyze the effect of the number of electrodes on the inverse solution/connectivity results. The montages used here are the 32, 64, 128 and hr-montage. The electrodes are selected based on the universal 5–5 system [67]. In the high-resolution montage, we excluded the electrodes located on the face as well as the few electrodes showing too high impedance. Overall, in this hr montage, 180 electrodes were found to provide excellent quality signals over all subjects (see Figure 2A where we present the 2D positions of the selected 180 electrodes).

Then, in step 3 the functional connectivity among cortical sources is computed according to a pair-wise approach using the methods described above (R^2^, sPLV, MI, ImC and PE). The time series of reconstructed sources are then filtered in the two frequency bands beta (14–30 Hz) and low gamma (30–45 Hz). Functional connectivity is calculated in these bands over the (>200 ms) time window that typically corresponded to the end of the recognition process and to the start of the naming process [54]. A thresholding procedure is applied on the functional connectivity values in order to retain a small fraction (10%) of the strongest functional connections, as discussed in [41].

Finally, in step 4, the performance of each method (source reconstruction+functional connectivity) is evaluated against its capacity to identify a network “topologically close” to that expected in the considered task. For this purpose, we proposed two criteria based on the definition of 7 (4 left and 3 right) distinct regions of interest (ROIs) reported to be involved in the cognitive task performed by the subjects. Table 1 provides detailed information about these ROIs.

**Table 1:** A summary of the previously published functional imaging studies of picture naming used to define the ROIs.

ROI	Location in the brain	Reference	Modality	BA
LO	Left	[48]	PET	17/19
	Occipital	[88]	fMRI	18/19
	lobe	[44]	PET	18
		[55]	Electrostimulation	–
		[42]	MEG/fMRI	–
		[89]	ECoG	–
RO	Right	[88]	fMRI	18/19
	occipital	[90]	fMRI	–
	lobe	[91]	fMRI	18/19
		[45]	PET/fMRI	18
		[42]	ECoG	–
LT	Left	[54]	MEG	–
	temporal	[91]	fMRI	37
	lobe	[92]	DWI/PWI	21/22/37/38
		[44]	PET	20/37/39
		[93]	PET	20
		[55]	Electrostimulation	–
		[89]	ECoG	–
RT	Right	[54]	MEG	–
	temporal	[90]	fMRI	–
	lobe	[94]	PET	–
		[92]	MEG	38
LF	Left	[44]	PET/fMRI	46
	frontal	[92]	DWI/PWI	44/45
	lobe	[42]	MEG/fMRI	–
		[95]	fMRI	–
RF	Right	[92]	DWI/PWI	44/45
	frontal	[46]	PET	–
	lobe	[95]	fMRI	–
LP	Left	[88]	fMRI	7
	parietal	[51]	MEG	–
	lobe	[42]	MEG/fMRI	–
		[89]	ECoG	–

**BA**: Brodmann Area, **fMRI**: functional Magnetic Resonance Imaging, **MEG**: Magnetoencephalography, **ECoG**: Electrocorticography, **PET**: Positron emission tomography, **DWI**: diffusion-weighted imaging, **PWI**: perfusion-weighted imaging.

The intuitive idea behind these two criteria is to quantify the extent to which the topology of an identified network fits with pre-defined ROIs. Qualitatively, a “correct” network is a network for which the significant connections involve sources distributed within and across these ROIs. Conversely, a network for which these connections only link sources outside these ROIs would reveal that the corresponding EEG signal processing is inappropriate.

The Localization Index (LI) is defined as the ratio between the number of connections identified inside all regions of interest (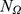) and the total number of identified connections:
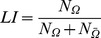
where 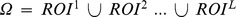 (*L* is the number of predefined ROIs) and 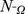 is the number of connections detected by the method *outside* these regions. This criterion is a “global” estimation of the performance of the inverse/connectivity combined approach. It is computed over the 7 pre-defined ROIs. The LI values range from 0 (poorest performance: all the edges are identified *outside* the ROIs) to 1 (highest performance: all the edges are identified *inside* the ROIs).

The second criterion (*R_i_*) represents the percentage of identified edges within each ROI*i*:
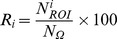
where *i* represents each of the 7 predefined ROIs (*i*: LO, RO, LP, LT, LF or RF, see figure 1). This criterion *R* is a “local” indicator about the distribution of identified edges in each ROI. This can be very important because the brain regions activated during the analyzed task are supposed to be distinct and because localization results are known to be dependent on the anatomical location of sources. *R_i_* varies from 0% (no edges are identified in the region *i*) and 100% (all the identified edges are localized in *i*).

### Factor analysis

In order to study the relationships between the three classes of factors (five connectivity measures, four inverse problem solutions and four EEG montages), we carried out a Multiple Factor Analysis (MFA) based on the Principal Component Analysis. This analysis was performed using XLSTAT software ©. The main goal is to understand how the different factors affect the LI values and which factor seems to be the most important one.

### High-resolution EEG Data

Twelve subjects were shown pictures (n = 148) on a screen using E-Prime 2.0 software (Psychology Software Tools, Pittsburgh, PA) [68]. They were asked to name the displayed pictures. The 148 images were selected from a database of 400 pictures standardized for French [69] and were used during two sessions (about eight minutes each) of 74 stimuli. The brain activity was recorded using a hr-EEG system (EGI, Electrical Geodesic Inc.). The unique feature of this system is the large coverage of the subject’s head by surface electrodes allowing for the improved analysis of the intracerebral activity from non-invasive scalp measurements, as compared with 32 -to 128- electrodes standard systems. EEG signals were collected with a 1 kHz sampling frequency and band-pass filtered between 3 and 45 Hz. Each trial was visually inspected, and epochs contaminated by eye blinking, movements or any other noise source were rejected and excluded from the analysis performed using the EEGLAB open source toolbox [70]. Statistical analyses of performance criteria were done using the Wilcoxon rank-sum test which is well suited for small sample sizes. To analyze the statistical differences in results obtained from tested combinations (inverse + connectivity), we used the Kruskal-Wallis test combined with a Bonferroni correction. The test was applied to the 20 possible combinations. This study was approved by the National Ethics Committee for the Protection of Persons (CPP), *conneXion* study, agreement number (2012-A01227-36), promoter: Rennes University Hospital). All participants provide their written informed consent to participate in this study. The ethics committee has approved the consent procedure.

## Results

**Figure 3:**
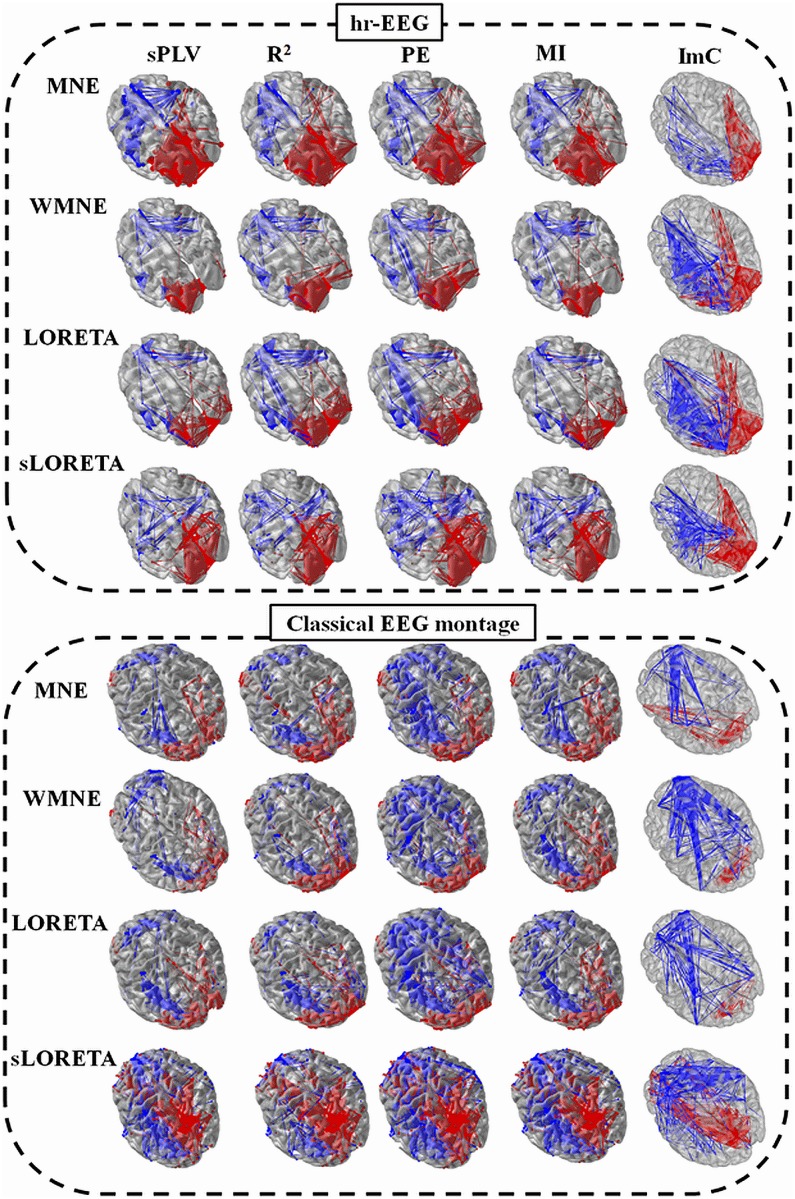
Connectivity graphs obtained by using the different inverse and connectivity methods for hr-EEG (Up) and classical EEG montage (Bottom). Red and blue lines denote the functional connectivity as measured in the gamma (>30 Hz) and beta (14–30 Hz) frequency band respectively.

A typical example of the connectivity graphs obtained for the 20 different combinations of the source reconstruction and functional connectivity methods is presented in figure 3, for the two montages (32 and hr). The qualitative visual inspection of the identified networks shows that results are highly dependent on the chosen algorithms used to solve the EEG inverse problem and to measure the functional connectivity. In addition, another source of variability comes from the number of electrodes retained for the analysis, as results also differ when the same combination (source reconstruction/connectivity estimate) is used either on classical or hr montage. In particular, when using 32 electrodes, the connections were found to be less dense especially in the occipital region (figure 3, upper box vs. lower box).

Regarding the connectivity measures, results were found to be fairly similar for a given inverse algorithm but strongly depend on the number of electrodes. Conversely, for a given brain connectivity method, the identified graphs strongly differed depending on the algorithm used to solve the inverse problem. For instance, using MNE with 180 electrodes (figure 3, upper box, first column), the sPLV, R^2^, PE, ImC and MI methods identified the same regions, but the activation in the right prefrontal region was more prominent when using the sPLV method. Qualitatively, and conversely to the four other methods, one can also notice that the graphs identified with ImC (whatever the inverse method) disclose a higher (resp. lower) number of long- (resp. short-) range connections.

Concerning the inverse algorithms, results showed higher variability as mentioned above. For instance, when using the sPLV method with 180 electrodes (figure 3, upper box, first line), MNE found an extended network in the left occipital lobe, as compared with the other methods. Along the same line, some regions identified with MNE, wMNE and LORETA, such as the right mesial frontal area, were not retrieved with sLORETA.

As far as the frequency band is concerned, the spatial distributions of the most significant edges computed either in the beta (24–30 Hz) or low gamma (30–45 Hz) bands were in relatively good agreement. Indeed, although discrepancies between graphs were evidenced depending on the methods employed, a general trend was found independently from the combination of algorithms: indeed, the highest gamma functional connectivity was obtained among sources located in the occipital lobe(s) while more significant beta connectivity was found among sources located in the other brain areas.

### Factor analysis

To quantify the qualitative observations reported in figure 3, a factor analysis was conducted. We summarize the whole analysis by describing only the interactions between the different factors/combinations and the variability of each factor. When analyzing the connectivity/inverse combination, we observed that the combinations with the same connectivity measure have lower correlation values (0.75 for R^2^/LORETA vs. R^2^/MNE and 0.8 for PE/MNE vs. PE/sLORETA) than combinations with the same inverse algorithm (0.97 for sPLV/wMNE vs. ImC/wMNE and 0.98 for MI/sLORETA and ImC/sLORETA) (i.e. stronger effect of inverse method). However, when analyzing the inverse problem/number of electrodes combination, the results showed that the correlations are relatively low for all the combinations (i.e. strong effect of both factors). We observed higher correlation values when changing the inverse method (0.54 for wMNE/32 vs. sLORETA/32 and 0.5 for LORETA/128 vs. MNE/128) than the number of electrodes (0.223 for wMNE/128 vs. wMNE/180 and 0.23 for MNE/128 vs. MNE/180). Regarding the connectivity/number of electrodes combination, results showed a low correlation when changing the number of electrodes (0.62 for ImC/64 vs. ImC/32 and 0.69 for PLV/64 vs. PLV/32) and slightly higher correlation when changing the connectivity method (0.79 for PE/180 vs. R^2^/180 and 0.8 for PE/64 vs. ImC/64) (i.e. stronger effect of number of electrodes).

Additionally, the variability of the LI values of each class of factors was then analyzed. The values showed that the EEG montages have the highest variability followed by the inverse problem algorithm and the connectivity measures. sPLV and ImC, 128 and 180 electrode montage and MNE/wMNE showed the highest effect among each class.

From these results, we can conclude that the connectivity measures have the lowest effect on the LI values; conversely to the number of electrodes that strongly impacts the LI values.

### Number of electrodes

**Figure 4:**
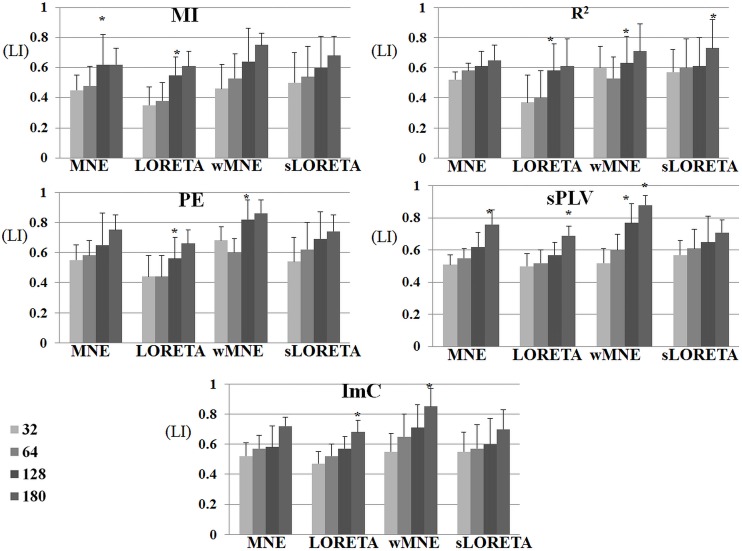
Comparison between the 32, 64, 128 and hr-montage for different inverse and connectivity methods. Asterisk above boxes indicates significant difference (*p*<0.05). **LI**: Localization Index.

Figure 4 reports the values of the performance criterion LI for the different inverse (MNE, LORETA, wMNE and sLORETA) and connectivity (sPLV, R^2^, PE, ImC and MI) methods, in the case of 32, 64, 128 channels and 180 channels EEG signals. As a general inspection, for all combinations of inverse/connectivity algorithms the LI values were higher for a large number of scalp EEG electrodes. The results of the Wilcoxon rank-sum test on the LI values (over subjects) are represented (* for *p*<0.05). The results don’t show any significantdifference between the 32 and 64 montages. A significant difference is obtained when increasing the number of electrodes up to 128 such as the case of MI/MNE (*p* = 0.021), R^2^/LORETA (*p*<0.01) and PE/LORETA (*p* = 0.04)/wMNE (*p*<0.01). The use of 180 channels has significantly increased the network identification in the case of R^2^/sLORETA (*p* = 0.021), sPLV/wMNE (*p* = 0.03)/LORETA (*p* = 0.022)/MNE (*p* = 0.02) and ImC/wMNE (*p* = 0.033)/LORETA (*p* = 0.34).

In addition, the results also indicated that the use of 32 or 64 electrodes only may provide a sub-estimation of the co-activated sources in the ROIs or/and an over-estimation of sources outside the ROIs, as revealed by the low LI values – obtained for some combinations such as LORETA/MI (LI = 0.35) and LORETA/R^2^ (LI = 0.37) - respectively. Figure 4 shows that the increase of the number of electrodes used in the source estimation improves the final result in term of identified brain networks at the source level. In addition, it also provides quantitative results regarding the differences between the inverse algorithms, for a given number of electrodes.

As depicted, for 180 electrodes, one can notice that wMNE combined with a phase synchronization method (sPLV) provided the highest average LI value (LI = 0.88). This result indicates that this combination (wMNE/sPLV) was able to localize 88% of significant connections distributed over the expected ROIs. Two other combinations (wMNE/ImC) and (wMNE/PE) also led to relatively high LI values (0.82 and 0.82, respectively). These values are in contrast with the poorer results obtained for LORETA/sPLV (LI = 0.69) and LORETA/PE (LI = 0.62) using the same number of electrodes (180). The results of the Wilcoxon rank-sum test indicates also that sPLV/wMNE is significantly different than sPLV/MNE (*p* = 0.035), sPLV/LORETA (*p* = 0.03), ImC/LORETA (*p* = 0.028), ImC/wMNE (*p* = 0.048) and R^2^/sLORETA (*p* = 0.034).

### Source reconstruction/functional connectivity combination

**Figure 5:**
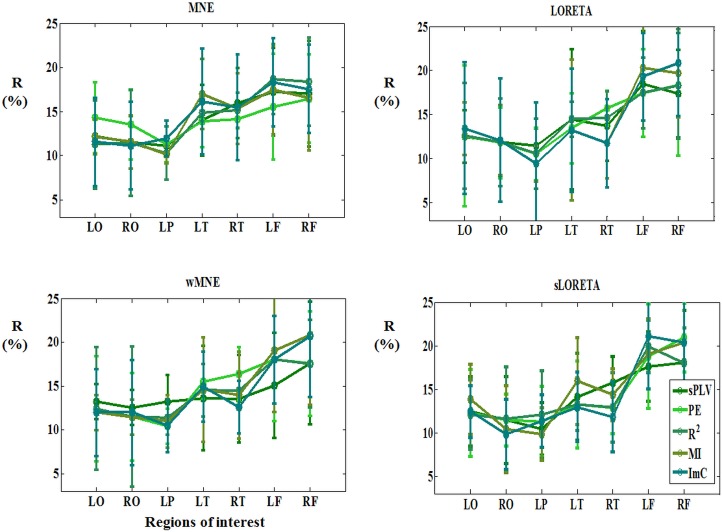
The mean and standard variations of the R (percentage of identified edges for within each ROI) values obtained for the different functional connectivity methods (computed over the 12 subjects).

In this section, we report the results obtained from the quantitative comparison of the performance of the inverse algorithms and connectivity methods, according to the R criterion (% of network connections in each ROI). In Figure 5, the R value curves obtained for the five connectivity methods are superimposed and plotted for each source reconstruction algorithm. Results suggest that all connectivity methods identified a comparable percentage of significant connections for all inverse methods. When the sLORETA algorithm was used, the MI method provided slightly different results than the other connectivity methods. Regarding the different ROIs, a higher performance of MNE, LORETA, wMNE and sLORETA was observed in the left than in the right hemisphere except the occipital part where we observe marginally higher values in the right hemisphere. For the ROI in the left parietal (LP) region, comparable R values were obtained for the five connectivity methods and for the inverse solution algorithms (MNE, WME and LORETA). Finally, the highest R values were obtained on ROIs located in the left frontal (LF) and the right frontal (RF) lobes. Interestingly, a trend in increasing R values was observed for most of the combinations (inverse/connectivity), suggesting that there may exist a posteroanterior gradient (occipito-temporo-frontal) of the density of connections within brain regions belonging to the network involved during picture recognition and naming.

### Typical example of brain networks obtained with the wMNE/sPLV combination

**Figure 6:**
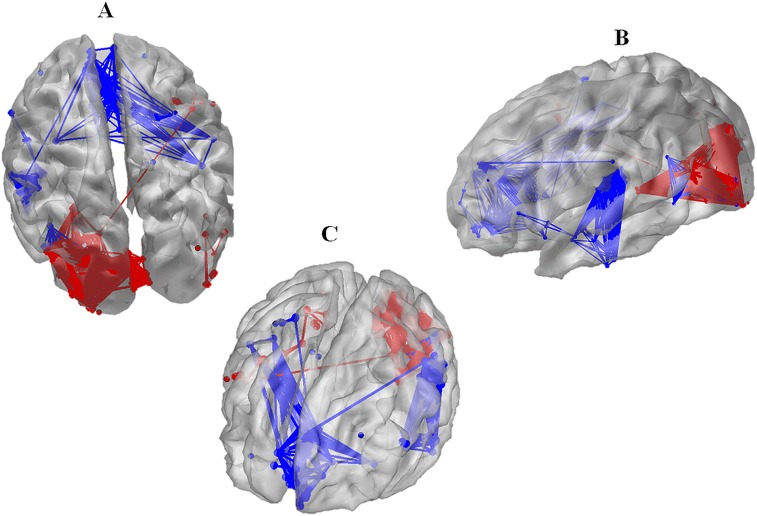
Typical example of the brain network identified using wMNE and sPLV in the picture recognition and naming task. A: lateral view B: Top view C: frontal view.

Table 2 provides the LI values computed for the 20 combinations of inverse solution and connectivity algorithms using 180 electrodes and averaged over the 12 subjects. The multiple comparisons between all combinations using the Kruskal-Wallis test combined with the Bonferroni correction method lead to conclude that the combination of wMNE/sPLV (with p = 0.05) has the best performance in term of topological correspondence between identified networks and ROIs defined from results reported in the literature for the same task (picture recognition and naming). In this part, we show a typical example of the identified networks obtained with this particular combination of methods. In figure 6A (top view), the functional network displayed in red color (as obtained when the sPLV method was applied to EEGs filtered in the gamma frequency band) is likely to correspond with the activation of the left visual areas (both primary and secondary). Figure 6B shows also an activated network in the right temporal gyrus. Figure 6B (left lateral view) shows that the identified networks involve the left inferior parietal region, the left superior temporal gyrus (posterior part) and the left inferior and middle temporal gyri. Finally, figure 6C (frontal view) indicates the presence of functional networks in the frontal pole (particularly the mesial part) with connections with the right parieto-temporal regions. We can observe also that the functional connections measured in the gamma frequency band (red) are more prominent in the occipital region while the other significant connections (within and across the parietal, temporal and frontal regions) were measured in the beta frequency band.

**Table 2:** Mean and standard deviations of LI values (over the 12 subjects) for the tested inverse and connectivity methods.

	MNE	LORETA	WMNE	sLORETA
MI	0.62±0.11	0.61±0.1	0.75±0.08	0.68±0.13
R2	0.65±0.07	0.61±0.12	0.71±0.03	0.73±0.07
PE	0.71±0.12	0.62±0.08	0.82±0.11	0.69±0.14
sPLV	0.76±0.09	0.69±0.06	**0.88±0.06**	0.71±0.08
ImC	0.68±0.08	0.63±0.06	0.82±0.1	0.69±0.13

## Discussion

The accurate identification, from noninvasive data, of brain networks related to specific cognitive functions is a major challenge. In this scope, EEG source localization techniques on the one hand and connectivity methods on the other hand have considerably developed over the past decades. However, the joint use of these two approaches is still recent and is recognized as relatively delicate regarding the number of methodological and technical issues that have to be solved to obtain relevant results. In this paper, we presented a novel comparative analysis of the results obtained from the possible combinations between four algorithms to solve the EEG inverse problem (estimating the extended sources) and five methods to estimate the functional connectivity. In addition, the influence of two key factors that intervene in both steps was also addressed, namely i) the number of electrodes used to solve the inverse problem and ii) the frequency bands retained to estimate the functional connectivity among neocortical sources. A general result from this comparative study is that rather different networks are identified from the same data when different combinations of methods are being used. Therefore, *cautiousness* is required regarding the (too) rapid interpretation of results. Nevertheless, some combinations identified brain networks concordant with those expected in the considered task, i.e. the picture recognition and naming. More specific discussions about the results are highlighted hereafter.

### Inverse/connectivity combination

We proposed a *joint* comparison of the inverse and connectivity methods, which has never been done so far. The results show that the choice of the inverse algorithm and the connectivity method is crucial and can strongly alter the results and their interpretation. It is remarkable that starting from the exact same EEG recordings, different networks can be identified. Results showed that the final result (i.e. the functional connectivity network identified at source level) strongly depends on the method that is chosen to solve the EEG inverse problem andon the functional connectivity method. By proposing a quantitative comparison procedure, we were able to retain one combination that showed the best performance in identifying the brain networks supposed to be activated during the picture recognition and naming task. Nevertheless, we have to mention that some other inverse algorithms (like MUSIC-based algorithms and beamforming) as well as some connectivity measures (like generalized synchronization and envelope based methods [23]) were not included in the study.

### The number of electrodes

The effect of the number of electrodes on the connectivity analysis has not received enough attention in the identification of cognitive networks underlying picture naming. Here, we tackled this issue by comparing activated sources in pre-defined regions of interest (those reported in the literature) by using either 32 or 180 electrodes. Our results show a clear improvement of the brain network identification when increasing the number of channels. Other montages (with 64 or 120 electrodes) could also be tested, but the overall message will remain the same. This finding is in agreement with a number of studies that already reported that source localization results can be improved when the number of electrodes is increased [8], [71]–[74].

### Connectivity at two different frequency bands

The frequency bands used to compute the functional connectivity is also a critical parameter as reported by [10], [18]. Our results suggest that significantly high correlation values are mainly found between oscillations in the beta band (14–30 Hz) generated at the level of relatively distant sources as well as oscillations in the low gamma band (30–45 Hz) but for closer sources, as typically observed in the occipital cortex. These results are in line with several studies suggesting that beta oscillations are related to long-range synchronization while gamma oscillations are more related to short range synchronization [75]–[77]. It is noteworthy that our quantitative comparison did not account for the other EEG frequency bands, as there is no solid “ground truth” about the functionality of the delta, theta and alpha bands in the considered task. In addition, we chose to restrict the gamma band to 30–45 Hz to avoid any contamination of connectivity measures by the power-line noise (50 Hz in France) or by any stop-band filtering effect.

### Identification of brain networks involved in picture recognition and naming

In the example provided at the end of the paper (Figure 6), we showed that using the best combination of inverse (wMNE)/connectivity (sPLV) methods, the optimal number (180) of electrodes and the two frequency bands (gamma and beta), we were able to identify networks topologically close (in term of involved brain networks) to that activated in picture recognition and naming task (as reported in the literature). A difficulty we faced in this study is that the regions activated during picture recognition and naming may differ among reported studies as based on different protocols and neuroimaging techniques. Nevertheless, the identified network includes three main regions that are commonly reported as being activated in the considered cognitive task: the bilateral occipital, the left temporal and the left/right frontal regions. It also includes the left parietal and right temporal which were less described in the literature. The detailed analysis showed that this identified functional network involves the left occipital region which is well known to play a capital role in the processing visual information [78], [79]. Results also revealed an implication of the right temporal gyrus which is characteristic of living pictures [80]. The other regions, namely the left inferior parietal region, the left superior temporal gyrus (posterior part), the frontal pole and the left inferior temporal middle temporal gyrus could also be identified by the wMNE/sPLV procedure. These regions were reported to be related, respectively, to the representation of objects, to working memory, to visual analysis/associations respectively and to working memory/memory retrieval [81]–[85]. Finally, a very dense network was found in the left inferior temporal lobe which has been recently reported as the “semantic hub” of the brain [86].

### Most important factors

In the paper, we achieved a MFA to investigate the impact of the different factors. We also analyzed the variability of each factor. Results showed that the EEG montages and the inverse problem methods have high impact on the results. However, the connectivity measures have lower effect. This MFA confirmed that cautiousness is needed in the choice of the inverse problem method and in the number of electrodes when performing EEG source connectivity analysis.

### Open issues

A classical and still unsolved difficult question relates to the setting of threshold values applied on the reconstructed sources as well on the connectivity measures. In this comparative study, the same threshold value was used for each combination of methods 10%, [41]. Other approaches can be explored like those based on surrogate data [62], although requiring a higher computation time. In future investigations, the influence of this parameter will be assessed in the MFA.

Another issue is related to the effect of field spread on the source connectivity analysis which has been reported by several recent studies (see [14]). The biggest challenge in EEG/MEG measures of functional connectivity is that the ill posed nature of the inverse problem means that spatially separate localized sources are not necessarily independent. This consideration led us to add the imaginary coherence (as reported in [38]) to our comparative study. Results showed some topological differences as compared with the other connectivity methods, especially in term of ratio between long- and short- range connections in the identified network. A more detailed investigation of these differences could be performed using some other approaches based on the use of beamformer and envelope connectivity analysis [87], for instance.

## Conclusion

EEG source connectivity can be a valuable method to identify brain networks underlying specific cognitive function. However, results are highly dependent on the choice of processing methods. In this study, we assessed the variability of results with respect to methodological issues in a cognitive task (picture recognition and naming) for which some “ground truth” can be put forward based on the collected literature on the topic. Our results suggest that the combination of wMNE and sPLV techniques applied to hr-EEG leads to a relevant result when compared to results reported in this same task. We have evaluated several inverse solutions and connectivity measures. Our results suggest that the estimation of the EEG source connectivity is a difficult issue and that one should be careful when studying functional connectivity at the level of reconstructed sources. Our findings show a high variability between methods. Thus, a main outcome of this study is a message of *cautiousness* when analyzing EEG source connectivity. An optimal combination of inverse/connectivity methods in additional to a large number of electrodes must be chosen to best identify brain networks involved in cognitive tasks and very likely during pathological processes.
